# Multi-Omics Analysis of NCI-60 Cell Line Data Reveals Novel Metabolic Processes Linked with Resistance to Alkylating Anti-Cancer Agents

**DOI:** 10.3390/ijms241713242

**Published:** 2023-08-26

**Authors:** Blake R. Rushing

**Affiliations:** 1 Nutrition Research Institute, University of North Carolina at Chapel Hill, Kannapolis, NC 28081, USA; blake_rushing@unc.edu; 2 Department of Nutrition, University of North Carolina at Chapel Hill, Chapel Hill, NC 27599, USA; 3 Department of Pathology and Laboratory Medicine, University of North Carolina at Chapel Hill, Chapel Hill, NC 27599, USA; 4 Lineberger Comprehensive Cancer Center, University of North Carolina at Chapel Hill, Chapel Hill, NC 27599, USA

**Keywords:** alkylating agents, drug resistance, cancer, metabolic reprogramming, multi-omics, metabolomics, transcriptomics, proteomics, CNV

## Abstract

This study aimed to elucidate the molecular determinants influencing the response of cancer cells to alkylating agents, a major class of chemotherapeutic drugs used in cancer treatment. The study utilized data from the National Cancer Institute (NCI)-60 cell line screening program and employed a comprehensive multi-omics approach integrating transcriptomic, proteomic, metabolomic, and SNP data. Through integrated pathway analysis, the study identified key metabolic pathways, such as cysteine and methionine metabolism, starch and sucrose metabolism, pyrimidine metabolism, and purine metabolism, that differentiate drug-sensitive and drug-resistant cancer cells. The analysis also revealed potential druggable targets within these pathways. Furthermore, copy number variant (CNV) analysis, derived from SNP data, between sensitive and resistant cells identified notable differences in genes associated with metabolic changes (WWOX, CNTN5, DDAH1, PGR), protein trafficking (ARL17B, VAT1L), and miRNAs (MIR1302-2, MIR3163, MIR1244-3, MIR1302-9). The findings of this study provide a holistic view of the molecular landscape and dysregulated pathways underlying the response of cancer cells to alkylating agents. The insights gained from this research can contribute to the development of more effective therapeutic strategies and personalized treatment approaches, ultimately improving patient outcomes in cancer treatment.

## 1. Introduction

Alkylating agents represent a pivotal class of chemotherapeutic agents extensively employed in the treatment of various cancers. These compounds exert their therapeutic effects by inducing DNA damage through the covalent modification of DNA bases, leading to DNA damage and subsequent cell death [1]. Although alkylating agents have shown remarkable efficacy, the response of cancer cells to these drugs is highly heterogeneous, with a subset of cells displaying resistance over the course of treatment [2]. Understanding the underlying mechanisms that govern the sensitivity or resistance of cancer cells to alkylating agents is crucial for the development of more effective therapeutic strategies and personalized treatment approaches. Numerous factors contribute to the differential response of cancer cells to alkylating agents, including the expression and activity of DNA repair enzymes, drug uptake and efflux mechanisms, cell cycle checkpoints, and alterations in apoptotic pathways [3]. Moreover, the interplay between these factors within the complex landscape of cancer cells further complicates the determination of the precise mechanisms underlying drug sensitivity or resistance.

Several mechanisms have been identified that contribute to cancer cells acquiring or their inherent resistance to alkylating agents. Alkylating agents can be metabolized by various enzymes, including glutathione S-transferases (GSTs) and aldehyde dehydrogenases (ALDHs), which can inactivate or detoxify the drugs. The increased expression or activity of these enzymes can lead to reduced intracellular drug concentrations, thereby limiting their cytotoxic effects [4]. Cancer cells that have enhanced DNA repair capacity, such as increased expression or activity of DNA repair enzymes like O6-methylguanine-DNA methyltransferase (MGMT), nucleotide excision repair (NER) proteins, or homologous recombination (HR) factors, can efficiently repair the DNA lesions induced by alkylating agents, promoting survival and resistance to therapy [5,6,7]. The activation of survival signaling pathways, such as PI3K/AKT and MAPK/ERK, can confer resistance to alkylating agents by promoting cell survival, DNA repair, and anti-apoptotic mechanisms [8,9,10]. Additionally, alterations in cell cycle checkpoints, such as enhanced G2/M checkpoint activation or dysregulation of cell cycle regulators like p53, can contribute to resistance by allowing cancer cells more time to repair the DNA damage induced by alkylating agents [11,12]. Cancer cells that exhibit defects in apoptotic signaling pathways, such as decreased expression or mutations in pro-apoptotic proteins (e.g., BAX, BAK, caspases) or increased expression of anti-apoptotic proteins (e.g., Bcl-2, Bcl-xL), can evade cell death and exhibit resistance to alkylating agents [13,14,15].

In addition to these processes, cancer endogenous metabolism plays a crucial role in drug resistance, and understanding its significance is essential for the development of effective therapeutic strategies. Metabolic reprogramming is a hallmark of cancer, characterized by altered nutrient utilization, energy production, and biosynthesis [16,17,18]. This rewiring of cellular metabolism enables cancer cells to sustain their proliferation, adapt to hostile microenvironments, and resist therapeutic interventions, including drug treatments [19,20]. This leads to the unique metabolic properties of cancers including aerobic glycolysis, increased fatty acid synthesis, increased rates of glutamine metabolism, and more. These processes have been linked to cancer drug resistance [21], however, this area is understudied compared to other genetic and signaling-pathway-driven mechanisms for many drug classes, including alkylating agents. Therefore, more research is needed to investigate how cancer cell metabolism interacts with other cellular processes to facilitate the response to anticancer agents.

To address this critical knowledge gap, the current study was conducted to elucidate the molecular determinants that influence the response of cancer cells to alkylating agents using data from the National Cancer Institute (NCI)-60 cell line screening program. The NCI-60 cell line screen program tests the response of 60 cancer cell lines to thousands of test compounds that span numerous mechanisms of action [22]. Additionally, public datasets exist that have molecularly profiled the baseline characteristics of these cell lines. To this end, omics profiles were linked with sensitivity to alkylating agents in order to better understand the underlying molecular processes that determine the response of cancer cells to this drug class. By employing a comprehensive approach integrating transcriptomic, proteomic, metabolomic, and single nucleotide polymorphism (SNP) data, the current study takes a multidimensional view of the sensitivity of cancer cells to alkylating agents. These data offer a holistic view of the molecular landscape, network-level interactions, and dysregulated pathways related to alkylating agent response, and enables the discovery of novel biomarkers and predictive signatures to enhance our understanding of the complex and heterogeneous nature of drug resistance. Ultimately, these insights can inform the development of more effective therapeutic strategies and improve patient outcomes in cancer treatment.

## 2. Results

**Figure 1:**
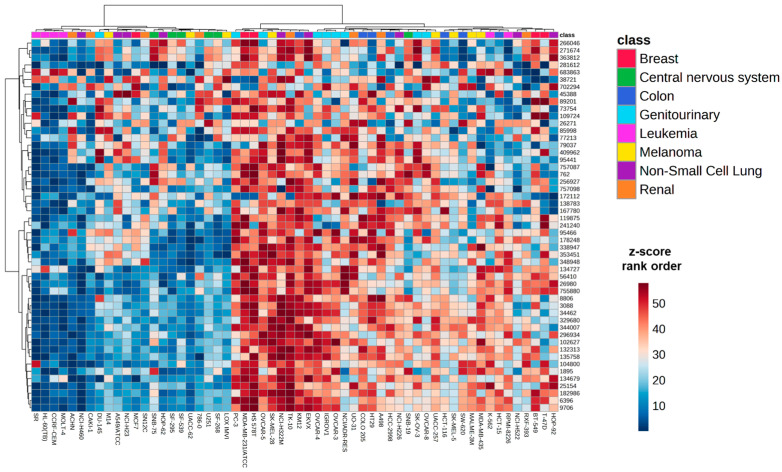
Heatmap of rank order of z-score for 51 alkylating agents across 58 cell lines in the NCI-60 cell line panel. Orange colors represent higher rank order values (more resistant) whereas blue colors represent lower rank order values (more sensitive). Distance measures were calculated using the Euclidean method and clustering was performed using the Ward method in MetaboAnalyst 5.0. Compounds are listed as NSC identifiers, which is an accession number given to each compound by NCI.

Mining NCI-60 cell line treatments revealed 51 chemical agents with an alkylating mechanism as defined by Cellminer. This included agents broadly defined as alkylating agents, as well as those that were specifically characterized as alkylating N-2, O-6, and N-7 positions of guanine residues (Supplemental Table S1). After converting z-scores to rank orders for each test compound, a clustering heatmap was generated using MetaboAnalyst (Figure 1). The unsupervised clustering of cell lines did not clearly indicate that sensitivity profiles to alkylating agents were driven by tissue type, although some tissue types such as leukemia and central nervous system cell lines tended to favor the lower z-score rank orders, indicating more sensitivity to these compounds.

**Figure 2:**
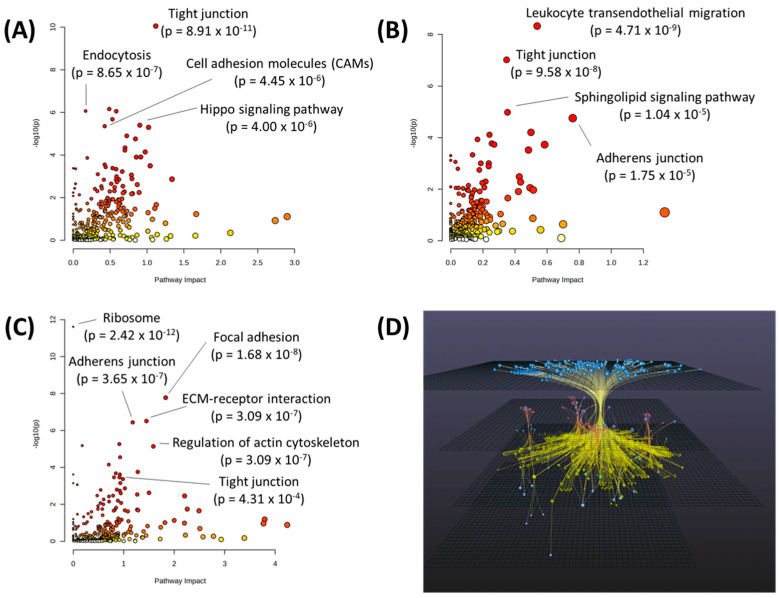
(**A**) Joint-pathway analysis of metabolites and transcripts with *p* < 0.05 between Q1 and Q4. (**B**) Joint-pathway analysis of metabolites and proteins with *p* < 0.05 between Q1 and Q4. (**C**) Pathway analysis of genes correlated to IC_50_ value of alkylating agents in the CREAMMIST database. Red points on the graph indicate a more significant pathway *p*-value. (**D**) Network analysis using metabolites, transcripts, and proteins with *p* < 0.05 between Q1 and Q4 of total rank order of z-scores. The network was built using Omicsnet and creating metabolite-protein interactions by mapping to the KEGG database. Grey nodes represent transcripts, red nodes represent proteins, yellow nodes represent metabolites, and red and grey nodes represent molecules present in both the transcriptomics and proteomics dataset. Nodes highlighted in blue represent seed nodes.

A total rank order of z-scores was calculated using the mean z-score value across all 51 alkylating agents for each cell line. This total rank order was used to divide the 58 cell lines into quartiles with the fourth quartile (Q4) having the highest overall z-score rank (most resistant to alkylating agents) and the first quartile (Q1) as having the lowest overall z-score rank (most sensitive to alkylating agents) (Table 1). Using these definitions, fold changes and *p*-values were calculated for all metabolites, transcripts, and proteins between Q1 and Q4 to identify the differentiators of cell lines that are resistant or sensitive to alkylating agents (Supplemental Table S2). This analysis revealed 34 metabolites, 4411 transcripts, and 530 proteins with a *p* < 0.05 between Q1 and Q4. These metabolites, transcripts, and proteins with *p* < 0.05 were input into MetaboAnalyst 5.0’s joint-pathway analysis to determine the cellular pathways that differentiated sensitive and resistant cells. Joint-pathway analysis of metabolites and transcripts identified the pathways associated with extracellular matrix (ECM) protein interactions (tight junction (*p* = 8.91 × 10^−11^), cell adhesion molecules) *p* = 4.45 × 10^−6^), and endocytosis (*p* = 8.65 × 10^−7^)) and the Hippo signaling pathway (4.00 × 10^−6^) (Figure 2A). Joint-pathway analysis of metabolites and proteins identified ECM-related pathways (leukocyte transendothelial migration (*p* = 4.71 × 10^−9^, tight junction (*p* = 9.58 × 10^−8^), and adherens junction (*p* = 1.75 × 10^−5^)) and the sphingolipid signaling pathway (*p* = 1.04 × 10^−5^) (Figure 2B). To add additional validation to these results, the CREAMMIST database was utilized which generates integrated pan-cancer dose-response curves across multiple publicly available studies and correlates drug IC_50_ values with gene expression patterns [23]. Ten alkylating agents were found in the CREAMMIST database (darbazine, procarbazine, bendamustine, oxaliplatin, mitomycin, chlorambucil, carboplatin, cyclophosphamide, cisplatin, and doxorubicin) and genes that were significantly correlated with > 50% of these drugs (Supplemental Table S3) were input into MetaboAnalyst 5.0 for pathway analysis. The results of this analysis also showed several ECM-related pathways, many of which were identified from the NCI-60 cell line data (adherens junction (*p* = 3.65 × 10^−7^), focal adhesion (1.68 × 10^−8^, ECM-receptor interaction (*p* = 3.09 × 10^−7^), and tight junction (4.31 × 10^−4^) (Figure 2C). This overlap in identified cellular pathways adds validity to the identified molecules that differentiate Q1 and Q4 cell lines. To narrow down a core set of molecules/pathways that differentiated Q1 and Q4, Omicsnet was utilized for an integrated network analysis using the KEGG database (Figure 2). This network analysis uses known biological relationships between each factor (proteins, transcripts, and metabolites) and builds a core set of molecules from input lists. This ensures that pathway analyses are enriched for molecules with logical connections to each other, removing outlier molecules without strong relationships to the entire set. The results showed that 71 pathways had a *p* < 0.05 (41 with an FDR-corrected *p*-value < 0.05) (a full list of pathway results for Figure 2 can be found in Supplemental Table S4). Four of the top five pathways were related to metabolic processes: cysteine and methionine metabolism (*p* = 4.36 × 10^−14^), pyrimidine metabolism (*p* = 5.02 × 10^−14^), starch and sucrose metabolism (*p* = 1.3 × 10^−12^), and purine metabolism (*p* = 1.34 × 10^−12^). EGFR tyrosine kinase inhibitor resistance was the top pathway differentiating Q1 and Q4 (*p* = 4.24 × 10^−73^) (Table 2).

**Figure 3:**
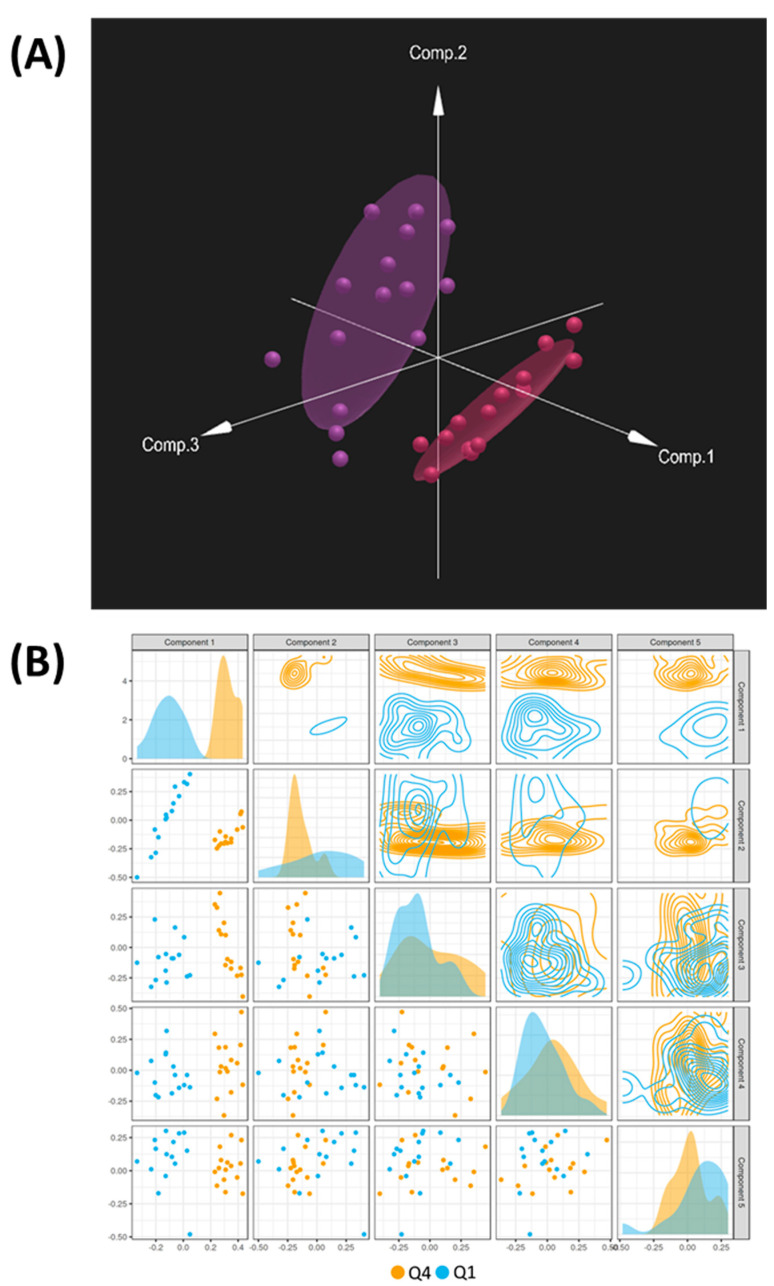
(**A**) DIABLO plot showing separation of Q1 (purple) and Q4 (red) using metabolomics, transcriptomics, proteomics, and SNP data. (**B**) Summary of first 5 components of the DIABLO model showing each component’s contribution to the separation of Q1 and Q4 samples.

**Figure 4:**
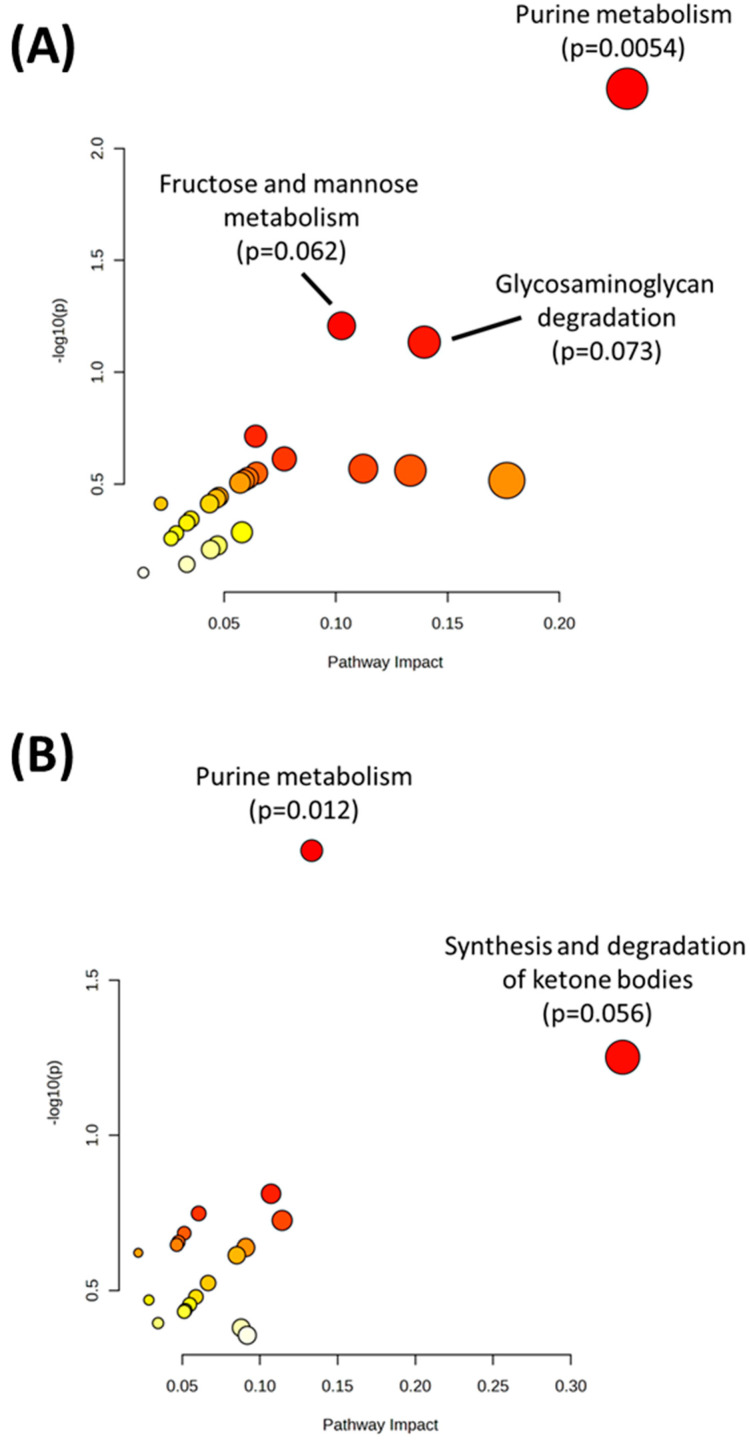
(**A**) Joint pathway analysis of metabolites and transcripts selected by DIABLO. (**B**) Joint pathway analysis of metabolites and proteins selected by DIABLO. Metabolic pathways were only selected for pathway analysis results. Red points on the graph indicate a more significant pathway *p*-value.

Multivariate analysis was performed using DIABLO to identify the complex relationship between the omics datasets and identify the strongest combination of predictors between Q1 and Q4. In addition to the metabolomics, transcriptomics, and proteomics datasets, SNP variant data were also uploaded to identify SNP variants that combined with the other omics datasets to differentiate Q1 and Q4. DIABLO analysis showed a clear separation between Q1 and Q4, with the first component of the model showing the strongest separation between the two groups (Figure 3A,B). Using this model, loadings scores were obtained for each variable (metabolite, transcript, protein, and SNP) and a cutoff of >|0.6| for the loadings values for the first component was used to identify significant variables. This resulted in 15 metabolites, 371 transcripts, 107 proteins, and 931 SNPs identified as differentiators (Supplementary Table S5). Joint pathway analysis using DIABLO-selected metabolites and transcripts (Figure 4A, Table 3) or metabolites and proteins (Figure 4B, Table 4) indicated that purine metabolism was the top significant pathway in both analyses. 

**Figure 5:**
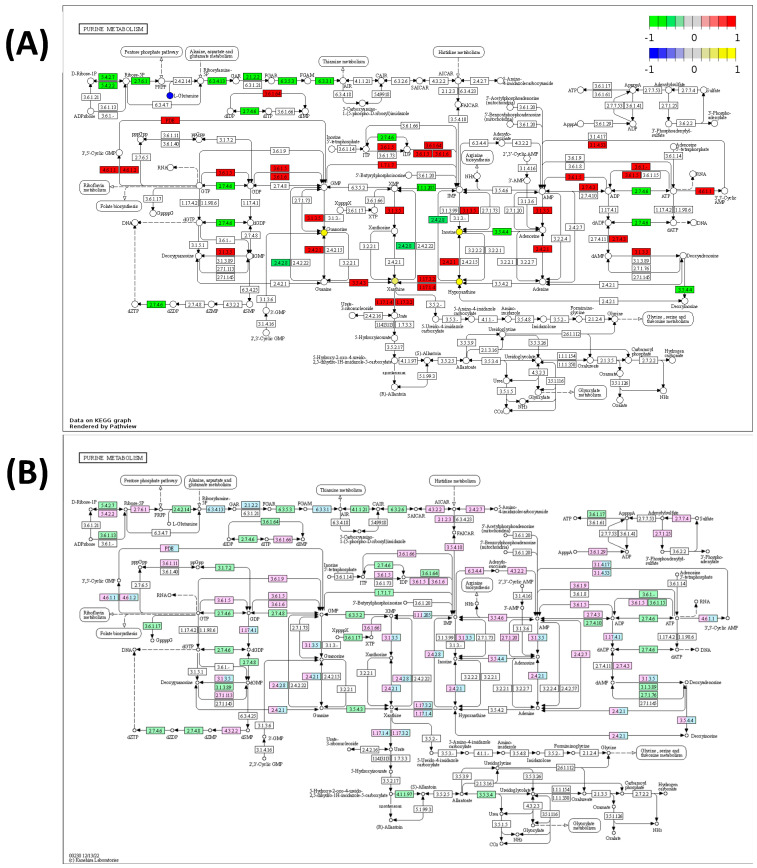
(**A**) Pathview analysis of the purine metabolism pathway using metabolites, transcripts, and proteins with *p* < 0.1 between Q1 and Q4. Genes in red are increased in Q4 whereas genes in green are decreased in Q4. Metabolites in yellow are increased in Q4 whereas metabolites in blue are decreased in Q4. (**B**) Purine metabolism KEGG map colored by druggable reactions. Genes colored in blue have at least one known drug which targets the corresponding enzymatic reaction. Genes colored in pink are associated with a disease. Genes colored in green are organism-specific genes.

Molecular differences in purine metabolism between Q1 and Q4 were visualized using Pathview with only metabolites, transcripts, or proteins with *p* < 0.1. Visualization showed decreases in the expression of enzymes in Q4 samples that control flux away from purine metabolism into other pathways, including the pentose phosphate pathway and amino acid pathways. Additionally, an increased expression of enzymes in the Q4 samples surrounding the base pair conversions was observed, and metabolites guanosine, xanthine, hypoxanthine, and inosine were also seen as increased in the Q4 samples in agreement with this observation (Figure 5A). Interestingly, the recorded doubling time of cell lines in Q4 is significantly higher than those of Q1, which may be related to this increase in nucleotide metabolism (cell line metadata including doubling time is included in Supplementary Table S6). An analysis of KEGG’s “Disease genes and drug targets” database revealed that many of these increased reactions in purine metabolism are known to be targeted by various drugs (Figure 5B), indicating the potential for the pharmacological targeting of these processes. 

CNV markers were also analyzed to uncover the potential genetic variants that may be contributing to these metabolic changes. DIABLO analysis selected 931 CNV markers that differentiated Q1 and Q4 (Supplementary Table S7). After matching CNV markers to their respective genes, 15 genes had >5 CNV markers identified as significantly different between Q1 and Q4. These genes included those that were increased in copy number intensity (MIR1302-2, MIR3163, CNTN5, MIR1302-9, DDAH1, DISC1FP1, PGR, and TRPC6) and those that were decreased in copy number intensity (MIR1244-3, WWOX, ARL17B, VAT1L, OR4F3, and FRG2) (Table 5).

**Figure 6:**
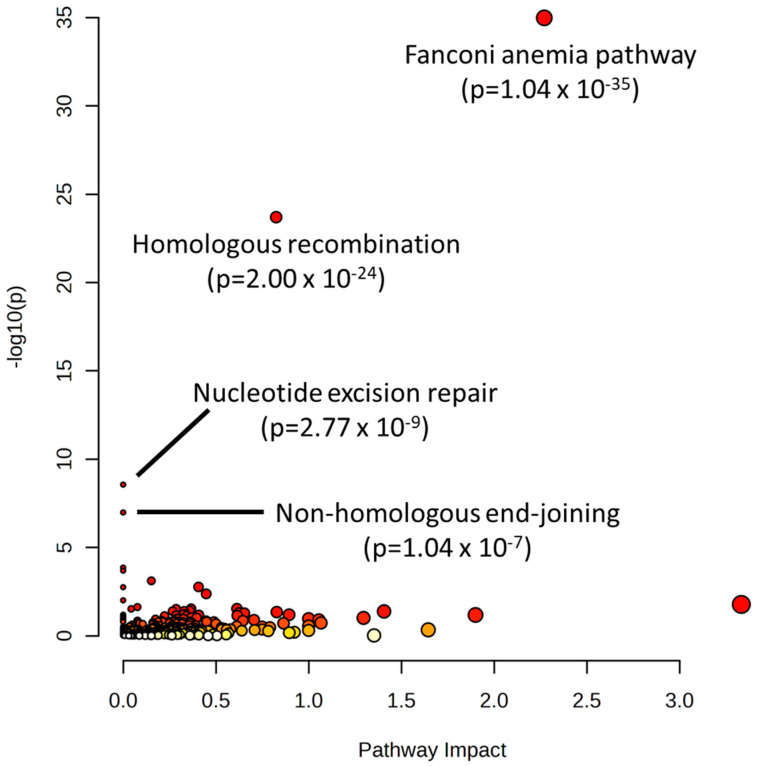
Pathway analysis of genes identified as hits for determining sensitivity to alkylating agents in the Biological General Repository for Interaction Datasets (BioGRID) Open Repository of CRISPR Screens (ORCS). Red points on the graph indicate a more significant pathway *p*-value.

Lastly, to confirm the importance of nucleotide metabolism in the sensitivity of cancer cells to alkylating agents, data were mined from the Biological General Repository for Interaction Datasets (BioGRID) Open Repository of CRISPR Screens (ORCS) [24]. Twelve CRISPR screen datasets were identified that investigated genes that modified sensitivity to various alkylating agents (cisplatin, doxorubicin, mitomycin, daunorubicin, oxaliplatin, and thiotepa). These datasets spanned four studies with >30,000 total genes screened [25,26,27,28]. All identified gene hits in these screens were input into MetaboAnalyst 5.0 for pathway analysis. The results showed pathways involved in repairing damaged nucleotides/DNA as highly significant in determining alkylating agent sensitivity: the Fanconi anemia pathway, (*p* = 1.04 × 10^−35^), homologous recombination (2.00 × 10^−24^), nucleotide excision repair (2.77 × 10^−9^), and non-homologous end-joining (1.04 × 10^−7^) (Figure 6). 

## 3. Discussion

The current study provides valuable insights into the molecular determinants of response to alkylating agents. Using a multi-omics approach of NCI-60 cell line data, we were able to assign multiple metabolic features that differentiated cancer cells that are sensitive or resistant to this class of anticancer agents. While pathways related to amino acid and carbohydrate metabolism were found to be significantly different, nucleotide metabolism—specifically purine metabolism—was found to be the largest metabolic differentiator between cells sensitive or resistant to alkylating agents. Overall, gene/protein expression and metabolite levels were increased in this pathway for resistant cells, especially for the purine salvage reactions involving the interconversion of guanosine, xanthine, hypoxanthine, and inosine. Additionally, our findings of several drug resistance pathways (e.g., EGFR tyrosine kinase inhibitor resistance, platinum drug resistance, and ABC transporters) provide validity to our analysis approach and support the linkage between these metabolic disruptions and drug resistance. 

The purine salvage pathway provides the majority of the cellular requirements for purines through the recycling of degraded bases to replenish the purine pool [29]. Interestingly, xanthine—which initiates the degradation pathway of purines [30]—was also increased in resistant cells. In addition to their role in balancing the purine pool, inosine, hypoxanthine, and xanthine can be incorporated into DNA when accumulated within the cell. This misincorporation of these bases into DNA is mutagenic, and has been shown to lead to point mutations [31,32]. Given that genomic instability has been linked to multi-drug resistance in various cancers, this alteration in purine metabolism may be a mechanism that is driving resistance to alkylating agents [33,34,35]. Indeed, certain enzymes involved in purine metabolism such as xanthine oxidoreductase (XOR) have been shown to play a role in linking the pathogenesis of cancers to metabolic disorders and obesity by increasing the inflammation and oxidative stress that facilitate transformation, proliferation, progression, and metastasis [36]. Our data also showed a decrease in glutamine, which is one of the substrates that contribute to the de novo biosynthetic pathway of purine metabolism [29], which may indicate a reliance on increased glutamine consumption to support these changes in nucleotide metabolism. Notably, several other studies have shown that purine metabolism is a metabolic vulnerability of cancer cells and that this pathway is implicated in therapy resistance in a number of cancer types [37,38,39,40,41,42,43,44,45]. The current study shows that purine metabolism is a differentiator for resistant cancer cells in an entire drug class, and that changes in certain aspects of this pathway (e.g., increases in xanthine- and hypoxanthine-related reactions) are the most robust differentiators. Moreover, these results are specific to alkylating agents as a class, whereas previous studies have looked at other drug classes or only at a specific alkylating agent drug. A better understanding of the linkages that drive drug resistance, such as purine metabolism dysregulation, have the potential to lead to therapeutic approaches for improving response to anticancer therapies.

An analysis of CNV data derived from SNP analysis identified differences in 15 genes between sensitive and resistant cells. Included in these genes was WWOX, a tumor suppressor gene that has been shown to regulate multiple metabolic processes, including glucose and lipid metabolism [46,47,48,49]. WWOX has also been shown to play a role in contributing to the drug resistance to cisplatin, one of the alkylating agents included in our analysis, in ovarian cancer [50]. Additionally, four microRNAs were identified as differentiators between sensitive and resistant cells. MicroRNAs have been shown to play significant roles in drug resistance, including alkylating agent drugs, through modifying the expression of genes involved in drug metabolism/uptake, cell cycle checkpoints, DNA repair, and others [51,52]. The microRNAs identified in this analysis—MIR1302-2, MIR3163, MIR1244-3, and MIR1302-9—have been studied to various degrees in the context of drug resistance. MIR3163 and MIR1244 expression has, interestingly, been shown to inhibit drug resistance in multiple studies [53,54,55]. The observation that these miRNAs have increased copy number intensity may indicate that the expression and utilization of drug-sensitizing miRNAs may be inhibited in resistant cells. Additionally, increased copy number can be linked to decreased gene expression (through increasing negative promoter activity, for example), and uncoupling gene expression from copy number has been observed in cancers, which may further explain our observation of increased copy number signal in these miRNAs in resistant cells [56,57,58]. The other omics datasets did not have information on these miRNAs, therefore the actual expression could not be verified. Regardless, targeting these potential inhibitory mechanisms may provide an interesting strategy to increase drug sensitivity in resistant cancer cells. Other miRNAs identified in our CNV analysis, MIR1302-2 and MIR1302-9, have not been well studied in the context of drug resistance and are candidates for future studies. Other genes identified in the CNV analysis were related to metabolic processes (DDAH1, PGR) and protein trafficking (VAT1L, ARL17B), which may be additional avenues for future studies to determine the relationship between genetic factors and drug resistance. Variation in these genes may play a role in pre-dispositioning cells to undergo the metabolic changes seen in purine metabolism and other pathways reflected in the metabolomics, transcriptomics, and proteomics datasets, providing a potential underlying mechanism for these pathway differences. Additionally, targeted approaches are a logical approach to follow up results from any untargeted omics study. Our investigation has identified purine metabolism as a pathway that plays a major role in the response of cancer cells to alkylating agents. Targeted methods designed to measure this pathway should be used in in vitro and in vivo experiments to confirm the metabolomic, transcriptomic, and proteomic changes seen in this study.

Multiple measures were taken to validate the results in this study. The first method involved the use of the CREAMMIST database to identify genes correlated with alkylating agent IC_50_ values and to identify their associated pathways. These identified pathways had a high degree of overlap with the NCI-60 results initially identified in the joint-pathway analysis of metabolites, transcripts, and proteins, with *p* < 0.05 between Q1 and Q4. The second method was the use of metabolites + transcripts and metabolites + proteins as separate joint-pathway analyses, both with the univariate and multivariate-selected molecules. Using this approach, agreement could be found between the two methods to identify pathways that were consistently identified as significant across the omics datasets. The third method was the combination of univariate and multivariate approaches, as well as the combination of pathway (metabolites + transcripts and metabolites + proteins) and network analysis approaches in the multi-omics analysis. Using these orthogonal approaches, commonalities in key pathways/molecules could be identified, allowing the results to be narrowed down. Lastly, additional validation was gained from analyzing data from BioGRID ORCS. CRISPR screens are an emerging method that allow for the identification of genes that significantly determine cancer cells’ ability to survive under a given selection pressure (e.g., drug treatment) [59]. An investigation of the CRISPR datasets studying alkylating agents revealed the DNA repair mechanisms directly related to nucleotide metabolism/usage, including the Fanconi anemia pathway, homologous and non-homologous recombination, and nucleotide excision repair. Indeed, the regulation of the nucleotide pool is a major mechanism by which cells regulate DNA repair [60]. Future research is needed to further understand the dynamics between nucleotide metabolism and alkylating agent response, which may reveal the biomarkers of sensitivity or novel mechanisms to improve drug response.

## 4. Materials and Methods

### 4.1. Acquisition of Public NCI-60 Cell Line Molecular Data

Publicly available omics datasets of baseline molecular characteristics of cell lines in the NCI-60 cell line panel were obtained. Metabolomics data (average of triplicate experiments) were downloaded from NCI’s Development Therapeutics Program (DTP) website. Proteomics data were downloaded from https://www.ebi.ac.uk/pride/archive/projects/PXD013615 (accessed on 25 May 2023) [61]. Averaged transcriptomics data were downloaded from https://www.ncbi.nlm.nih.gov/sites/GDSbrowser?acc=GDS4296 (accessed on 24 May 2023) [62,63]. Copy number variant (CNV) data, derived from SNP measurements, were downloaded from CellMiner (DNA Affy 500k CRMAv2) [64]. Two cell lines—MDA-N and SK-MEL-2—were removed from all datasets due to being absent in the metabolomics data. 

### 4.2. Compilation of Drug Response Data for Alkylating Agents in the NCI-60 Cell Line Panel

Drug activities for compounds tested in the NCI-60 cell line panel were downloaded from CellMiner in the form of z-scores—a transformed value that is calculated by taking mean-centered GI_50_ values and then dividing by the standard deviation of a given test compound across all cell lines [64]. Compound mechanism data were also downloaded from CellMiner to identify test compounds with alkylating mechanisms. Only compounds that were indicated to have experimental data following quality control review from NCI were used for the analysis. Z-scores for each alkylating test compound were converted to a rank order (without breaking ties) across cell lines, and the median rank order across all test compounds was calculated for each cell line to derive a total rank order for alkylating compounds. MetaboAnalyst 5.0 was used to generate heatmaps of z-scores [65]. 

### 4.3. Univariate Statistics, Pathway Analysis, and Network Analysis of Selected Features

Fold changes were calculated between cell lines in the top (Q4) and bottom (Q1) quartile of the total rank order of z-scores using group averages for each variable. *p*-values were calculated between Q1 and Q4 using Students’ *t*-test. Metabolites, proteins, and transcripts that had a *p*-value < 0.05 between Q1 and Q4 of the total rank order of z-scores were used for joint pathway analysis in MetaboAnalyst 5.0 [65]. Pathway hits were validated using data from the CREAMMIST database for cancer drug response prediction (https://creammist.mtms.dev/ (accessed on 26 July 2023) [23]. Ten alkylating agents were identified in the CREAMMIST database and genes significantly correlated (Spearman rank) with each drug’s IC50 value were identified. Genes that were significant in ≥50% of the ten alkylating agents were input into MetaboAnalyst’s pathway analysis. For network analysis, metabolites, proteins, and transcripts with *p* < 0.05 between Q1 and Q4 were input into Omicsnet [66]. Metabolites were entered as KEGG IDs, proteins were entered as Uniprot IDs, and transcripts were entered as official gene symbols. For database selection for metabolomics, transcriptomics, and proteomics datasets, KEGG was used to map metabolite–protein interactions. After generating the network, pathway analysis was conducted in Omicsnet using the KEGG (gene/protein) database. 

### 4.4. Multi-Omic Multivariate Analysis and Joint Pathway Analysis

Metabolomics, transcriptomics, proteomics, and SNP datasets were imported into Omicsanalyst for integrated analysis [67]. Due to data size constraints, only the top 25% of CNV markers by variance across all cell lines were imported. All datasets were autoscaled to provide similar distribution patterns. Integrated analysis was performed using Data Integration Analysis for Biomarker discovery using Latent variable approaches for Omics studies (DIABLO) [68]. DIABLO is a supervised multivariate method (multi-block PLS-DA), and was used to discriminate cell lines in Q1 and Q4 of the total rank order of z-scores for alkylating agents. Joint pathway analysis was performed using Metaboanalyst 5.0 using variables that had an absolute loadings value > 0.6 for the first principal component [65].

### 4.5. CNV Analysis

CNV markers that had an absolute loadings value > 0.6 for the first DIABLO principal component were aligned with associated genes indicated by CellMiner. To determine genes with increased or decreased CNV intensity in resistant cells, average intensity values were averaged for each marker in Q1 and Q4 samples, and all markers mapped to the same gene were summed. Genes with a summed intensity that was higher in Q4 were defined as increased whereas genes with a summed intensity that were lower in Q4 were defined as decreased. Frequency of mapped CNV markers for each gene was calculated to determine genes that had the greatest number of CNV markers that differentiated Q1 and Q4.

### 4.6. Integrative Pathway Visualization

To visualize pathway perturbations using multi-omic data, metabolites, proteins, and transcripts were input into Pathview with average values in Q1 and Q4 of total rank order of z-scores [69,70]. Only variables with a *p* < 0.1 were input into the analysis. hsa-Homo sapiens was chosen for the species database, and positive fold changes indicate an increase in Q4 of total rank order of z-scores (most resistant to drug treatment). KEGG’s disease gene and drug targets database was analyzed to determine druggable targets that overlapped with Pathview data [71].

### 4.7. Data Mining and Pathway Analysis of CRISPR Screen Hits of Alkylating Agents

Multi-omics analysis results were validated by searching for gene hits that determine alkyalting agent sensitivity in the Biological General Repository for Interaction Datasets (BioGRID) Open Repository of CRISPR Screens (ORCS) (https://orcs.thebiogrid.org/ (accessed on 26 July 2023)) [24]. A total of 11 datasets were identified that observed gene hits that modified alkylating agent sensitivity (1-PMID32649862, 6-PMID32649862, 14-PMID32649862, 15-PMID32649862, 23-PMID32355287, 27-PMID32355287, 33-PMID32355287, 11-PMID32355287, 1-PMID35221331, and 10-PMID34049503, 14-PMID34049503). Gene hits from all studies were input into MetaboAnalyst 5.0 for pathway analysis to determine pathways that were linked with alkylating agent sensitivity. 

## 5. Conclusions

In conclusion, we have taken an unbiased, multi-omics approach to identify molecular networks that differentiate cancer cells that are sensitive or resistant to alkylating agents. Our analysis revealed several metabolic processes that differentiated sensitive and resistant cells, with purine metabolism emerging as the major metabolic differentiator, as determined by a combination of univariate and multivariate approaches. Additionally, CNV analysis revealed a collection of genes which may play a role in establishing these metabolic phenotypes that differentiate response status to alkylating agents. It is currently unclear if these metabolic processes play a role in cancer cell response to other drug classes. Therefore, future research should be performed to use multi-omics approaches to identify the cellular pathways associated with drug response to other classes in an unbiased manner. Notably, our findings did not indicate that cancer cell type was a major driver of sensitivity to this drug class, suggesting that omics profiles would be a better indicator of drug responsiveness. Overall, this information provides new insights into the cellular processes that may be targeted to potentially prevent or overcome resistance to alkylating agents. Due to the metabolic nature of these differences, targeting these processes should be investigated pharmacologically and nutritionally. Indeed, nutritional factors have been shown to have anticancer activity, as well as chemosensitization properties, through targeted cancer cell metabolism, such as fatty acid beta oxidation, glucose metabolism, polyunsaturated fatty acid metabolism, or nucleotide metabolism, to name a few [72,73,74,75]. As such, nutritional intervention may provide an additional route to combat drug resistance. Further research is needed in this area to develop multiple approaches to improve the efficacy of alkylating agents in cancer therapy.

## Figures and Tables

**Table 1:** Median z-score rank orders of each cell line for alkylating agents in the NCI-60 cell line screen.

Cell Line	Cancer Type	Median z-Score Rank Order	Quartile Number
SR	Leukemia	3.5	1
HL-60(TB)	Leukemia	4	1
CCRF-CEM	Leukemia	6	1
MOLT-4	Leukemia	6	1
NCI-H460	Non-Small Cell Lung	6	1
UACC-62	Melanoma	8	1
ACHN	Renal	10	1
CAKI-1	Renal	11	1
SF-295	Central nervous system	12	1
SF-539	Central nervous system	14	1
LOX IMVI	Melanoma	15	1
786-0	Renal	15	1
MCF7	Breast	17	1
HOP-62	Non-Small Cell Lung	18	1
SN12C	Renal	19	1
SF-268	Central nervous system	20	2
U251	Central nervous system	20	2
A549/ATCC	Non-Small Cell Lung	20	2
NCI-H23	Non-Small Cell Lung	21	2
NCI-H522	Non-Small Cell Lung	22.5	2
SNB-75	Central nervous system	23	2
M14	Melanoma	23	2
SK-MEL-5	Melanoma	25	2
SW-620	Colon	26	2
HOP-92	Non-Small Cell Lung	26	2
DU-145	Genitourinary	26	2
HCT-116	Colon	29	2
RPMI-8226	Leukemia	30	2
RXF-393	Renal	30	2
MALME-3M	Melanoma	32	3
OVCAR-8	Genitourinary	32	3
T-47D	Breast	33.5	3
HCC-2998	Colon	34	3
BT-549	Breast	34.5	3
SNB-19	Central nervous system	35	3
K-562	Leukemia	35	3
NCI-H226	Non-Small Cell Lung	35.5	3
SK-OV-3	Genitourinary	35.5	3
IGROV1	Genitourinary	36	3
NCI/ADR-RES	Genitourinary	36	3
HCT-15	Colon	37	3
COLO 205	Colon	38	3
UACC-257	Melanoma	38	3
OVCAR-3	Genitourinary	39	4
UO-31	Renal	39	4
HT29	Colon	40	4
MDA-MB-435	Melanoma	40	4
A498	Renal	42	4
PC-3	Genitourinary	44	4
OVCAR-4	Genitourinary	45	4
OVCAR-5	Genitourinary	45	4
KM12	Colon	47	4
SK-MEL-28	Melanoma	47	4
EKVX	Non-Small Cell Lung	48	4
MDA-MB-231/ATCC	Breast	51	4
HS 578T	Breast	51	4
NCI-H322M	Non-Small Cell Lung	53	4
TK-10	Renal	53	4

Missing z-scores indicate that the corresponding cell line did not have z-score information for a given compound. A lower rank order indicates a higher z score (greater sensitivity to a compound). Compounds that did not have experimental data after quality control were removed. Ties were not broken for cell lines that had the same z-score for a given test compound.

**Table 2:** Top ten pathways by *p*-value from multi-omic pathway analysis using variables with *p* < 0.05 between Q1 and Q4.

Pathway	*p*-Value	FDR
EGFR tyrosine kinase inhibitor resistance	4.24 × 10^−73^	1.42 × 10^−70^
Cysteine and methionine metabolism	4.36 × 10^−14^	5.62 × 10^−12^
Pyrimidine metabolism	5.02 × 10^−14^	5.62 × 10^−12^
Starch and sucrose metabolism	1.3 × 10^−12^	9.03 × 10^−11^
Purine metabolism	1.34 × 10^−12^	9.03 × 10^−11^
ABC transporters	7.98 × 10^−09^	4.47 × 10^−07^
Nicotinate and nicotinamide metabolism	9.81 × 10^−09^	4.71 × 10^−07^
Alanine, aspartate, and glutamate metabolism	9.69 × 10^−08^	4.07 × 10^−06^
Valine, leucine, and isoleucine degradation	1.91 × 10^−07^	7.12 × 10^−06^
Platinum drug resistance	1.21 × 10^−06^	4.07 × 10^−05^

**Table 3:** Top ten pathways from joint pathway analysis of metabolites and transcripts selected by DIABLO.

Pathway	*p*-Value
Purine metabolism	0.005412
Fructose and mannose metabolism	0.062016
Glycosaminoglycan degradation	0.073378
Amino sugar and nucleotide sugar metabolism	0.193000
Arginine biosynthesis	0.243890
Pyrimidine metabolism	0.269520
One carbon pool by folate	0.274760
Pentose and glucuronate interconversions	0.282290
Pantothenate and CoA biosynthesis	0.297110
Selenocompound metabolism	0.304420

**Table 4:** Top ten pathways from joint pathway analysis of metabolites and proteins selected by DIABLO.

Pathway	*p*-Value
Purine metabolism	0.012062
Synthesis and degradation of ketone bodies	0.056019
Butanoate metabolism	0.154510
Pantothenate and CoA biosynthesis	0.178800
Terpenoid backbone biosynthesis	0.188330
Fructose and mannose metabolism	0.207080
Starch and sucrose metabolism	0.220880
Beta-Alanine metabolism	0.225430
Pyruvate metabolism	0.229960
Biosynthesis of unsaturated fatty acids	0.238940

**Table 5:** Top genes identified as significantly different between Q1 and Q4 based on CNV data selected by DIABLO.

Gene Symbol	Gene Name	Number of CNV Markers	Direction of Change in Q4
MIR1302-2	microRNA 1302-2	149	Increased
MIR3163	microRNA 3163	87	Increased
MIR1244-3	microRNA 1244-3	27	Decreased
WWOX	WW domain containing oxidoreductase	22	Decreased
CNTN5	contactin 5	17	Increased
ARL17B	ADP ribosylation factor like GTPase 17B	12	Decreased
VAT1L	vesicle amine transport 1 like	10	Decreased
OR4F3	olfactory receptor family 4 subfamily F member 3	9	Decreased
MIR1302-9	microRNA 1302-9	8	Increased
DDAH1	dimethylarginine dimethylaminohydrolase 1	7	Increased
DISC1FP1	DISC1 fusion partner 1	7	Increased
PGR	PGR	7	Increased
RNU6-2	RNA, U6 small nuclear 2	7	Increased
FRG2	FSHD region gene 2	6	Decreased
TRPC6	transient receptor potential cation channel subfamily C member 6	6	Increased

Only genes with >5 CNV markers with a DIABLO loadings value > |0.6| are displayed. Direction of change for each gene was determined by comparing the sum of all marker intensities in Q4 compared to Q1.

## Data Availability

All data can be found in the Supplementary Material and the provided links to public datasets.

## Supplementary Materials

The supporting information can be downloaded at: https://www.mdpi.com/article/10.3390/ijms241713242/s1.
